# The correlation between rheological properties and extrusion-based printability in bioink artifact quantification

**DOI:** 10.1016/j.matdes.2023.112237

**Published:** 2023-08-12

**Authors:** Gregory J. Gillispie, Joshua Copus, Meryem Uzun-Per, James J. Yoo, Anthony Atala, Muhammad Khalid Khan Niazi, Sang Jin Lee

**Affiliations:** a Wake Forest Institute for Regenerative Medicine, Wake Forest University School of Medicine, Winston-Salem, NC 27157, USA; b School of Biomedical Engineering and Sciences, Wake Forest University-Virginia Tech, Winston-Salem, NC 27157, USA; c Center for Biomedical Informatics, Wake Forest University School of Medicine, Winston-Salem, NC 27157, USA

**Keywords:** 3D bioprinting, Bioink, Hydrogel, Printability, Rheology, Microextrusion

## Abstract

Bioinks for cell-based bioprinting face availability limitations. Furthermore, the bioink development process needs comprehensive printability assessment methods and a thorough understanding of rheological factors’ influence on printing outcomes. To bridge this gap, our study aimed to investigate the relationship between rheological properties and printing outcomes. We developed a specialized bioink artifact specifically designed to improve the quantification of printability assessment. This bioink artifact adhered to established criteria from extrusion-based bioprinting approaches. Seven hydrogel-based bioinks were selected and tested using the bioink artifact and rheological measurement. Rheological analysis revealed that the high-performing bioinks exhibited notable characteristics such as high storage modulus, low tan(δ), high shear-thinning capabilities, high yield stress, and fast, near-complete recovery abilities. Although rheological data alone cannot fully explain printing outcomes, certain metrics like storage modulus and tan(δ) correlated well (R^2^ > 0.9) with specific printing outcomes, such as gap-spanning capability and turn accuracy. This study provides a comprehensive examination of bioink shape fidelity across a wide range of bioinks, rheological measures, and printing outcomes. The results highlight the importance of considering the holistic view of bioink’s rheological properties and directly measuring printing outcomes. These findings underscore the need to enhance bioink availability and establish standardized methods for assessing printability.

## 1. Introduction

The field of bioprinting is making significant strides toward the fabrication of intricate tissue structures [1,2]. However, a major road-block hindering progress is the scarcity of suitable biomaterials called bioinks. Bioinks are combinations of hydrogels, cells, and/or signaling molecules that dictate the flow and mechanical properties of the bioinks [3]. The requirements imposed on bioinks can be broadly categorized into two aspects: biological properties and printability [4–6]. While various measures have been established and standardized to evaluate the biological properties of bioinks, including cell viability/proliferation assays, and histological and immunohistochemical analyses, the development of measures specifically focusing on bioink printability remains limited [4].

Printability refers to the performance of a bioink, specifically during the printing process [4,7]. Despite its significance, the evaluation of bioink printability is often described qualitatively in research reports, lacking reproducible or reliable quantitative measures. Consequently, there is a need for direct measures that can assess the printability of bioinks in a specific and standardized manner, ensuring their suitability for various bioprinting applications. Although a few measures have been developed, such as structural integrity [8], overhang collapse [9], and *Pr* value [10], each of these measures only captures a limited aspect of bioink behavior. Since bioinks must be capable of creating diverse structures, there is a pressing need for a comprehensive and bioink-specific methodology to evaluate printability and aid in the advancement of bioink development.

Rheological measurement has emerged as a widely adopted approach to examine the influence of different rheological factors on bioink printability. Notably, it has proven successful in assessing the extrudability of materials, where shear-thinning models have reliably predicted the pressure-flowrate relationships of various bioinks [11–14]. Within the rheological framework, several parameters, including viscosity, storage modulus, loss modulus, tan(δ), yield stress, and recovery capabilities, have been linked to the final printing outcomes [8–11,15]. However, the existing studies have been limited in their scope, typically focusing on a single rheological parameter using a specific model bioink, often at different concentrations, and with a single measurement of printing outcome. This limited approach may restrict the generalizability of the findings to other bioinks. Moreover, the interrelationships between different rheological measures have further complicated the matters. For instance, increasing the hydrogel concentration often leads to higher values of viscosity, storage modulus, loss modulus, yield stress, and improved shape fidelity.

Our previous work involved the development of a comprehensive printability framework utilizing gelatin-alginate hydrogel blends at varying concentrations [8]. We investigated the influence of dynamic modulus, including storage modulus (G’), loss modulus (G“), and loss tangent (G”/G’), on printing outcomes. The results demonstrated that a lower loss tangent correlated with improved structural integrity, whereas a higher loss tangent enhanced extrusion uniformity. Gelatin-alginate hydrogels within a loss tangent range of 0.25–0.45 exhibited an optimal balance between structural integrity and extrusion uniformity. However, it is essential to recognize that this range may differ for other hydrogel-based bioinks. Furthermore, other studies have investigated the evaluation of printability in bioinks through the use of rheological measurements. One study focused on the time- and temperature-evolving chitosan hydrogels [16], while another employed a machine-learning approach to predict the printability [17]. However, these investigations were limited in terms of the bioink formulations tested and the scope of rheological properties analyzed, as well as the simplicity of the printed pattern analysis. Nevertheless, further research is needed to encompass a wider range of bioink formulations and employ more comprehensive rheological analyses to advance our understanding and predictive capabilities in bioink development.

To expedite the progress of novel bioink development, a comprehensive understanding of the relationship between the rheological properties and printing outcomes is crucial. However, previous studies aiming to predict the outcomes of printed structures have yielded inconclusive results, implicating a multitude of rheological factors that need more practical applicability. Due to the incomplete understanding of the connections between rheology and other aspects of printability, rheology cannot currently serve as a reliable proxy for assessing printability. Therefore, the objective of this study is to investigate the correlation between rheological properties and printing outcomes by utilizing a novel bioink artifact quantification. The bioink artifact serves as a valuable tool to examine the correlation between bioinks with diverse rheological characteristics and the resulting printing outcomes. By employing this approach, we aim to shed light on the intricate interplay between rheology and printability, ultimately facilitating the development of more effective bioinks for bioprinting applications.

## 2. Materials and methods

### 2.1. Bioink preparation

Alginate (A2033), gelatin (G6144), gellan gum (G1910), glycerol (G6279), hyaluronic acid (53747), methylcellulose (M0262), and Pluronic F127 (P2443) were purchased from (Millipore Sigma, St. Louis, MO). Laponite RD and Laponite EP were purchased from BYK Additives and Instruments (Gonzales, TX). Gelatin methacrylate (GelMA) was synthesized as described previously [19]. Briefly, methacrylate anhydride was added at 0.5 mL/min for 3 h to a solution of gelatin type A at 50 ^◦^C. The solution was then diluted with PBS, dialyzed in distilled water, and lyophilized before storage at −20 ^◦^C. All materials were dissolved in UltraPure Distilled water (Invitrogen by Life Technologies, Grand Island, NY) and equilibrated for 10 min in the printing syringe at 21 ^◦^C prior to printing unless otherwise noted.

Pluronic F127 (PF) was dissolved at 40% w/v on a shaker at 4 ^◦^C for 24 h. Afterward, the PF was removed from the shaker, and the formed bubbles were allowed to settle before use. For alginate-laponite mixtures, 1% w/v alginate and either 6% w/v laponite RD (Alg-Lap-RD) or 6% w/v laponite EP (Alg-Lap-EP) were added to a 10 mL syringe and stirred with a plastic spatula to break up the largest chunks. After stirring, the syringe contents were mixed thoroughly by repetitively transferring them back and forth to another syringe using a syringe connector. After 1 h, syringe contents were mixed again and then centrifuged to remove air bubbles. For the gellan gum-GelMA formulation (GG/GM), 1.2% w/v gellan gum and 10% v/v glycerol were dissolved in an 80 ^◦^C oil bath under magnetic stirring for 15 min. The bath was then changed to 60 ^◦^C and 4% w/v GelMA was added and stirred for 20 min or until fully dissolved. The formulation was then filtered through 0.45 μm Whatman syringe filters (GE Healthcare Life Sciences, Marlborough, MA) which were pre-warmed to prevent rapid gelation within the filter. After transferring to the printing syringe, the formulation was placed in ice water for 5 min and then equilibrated for 10 min at 21 ^◦^C. For the alginate-only bioink (ALG), alginate was added to a 10 mL syringe at 7% w/v and stirred with a plastic spatula to break up the large chunks. After stirring, the syringe contents were mixed thoroughly by repetitively transferring them back and forth to another syringe using a syringe connector. After 1 h, syringe contents were mixed again and then centrifuged to remove air bubbles. For methylcellulose (MC) only, MC was added at 8% w/v slowly to DPBS (HyClone Laboratories, GE Healthcare Life Sciences) at 55 ^◦^C under magnetic stirring for 20 min. The material was then removed from heat but remained under magnetic stirring until reaching room temperature. It was subsequently placed at 4 ^◦^C for 24 h to dissolve prior to use fully. Lastly, for hyaluronic acid (HA) only, HA was added at 3% w/v to a 10 mL syringe and mixed vigorously back and forth to another syringe using a syringe connector. The material was then dissolved overnight at 37 ^◦^C on a shaker, mixed again through the syringe connector, and centrifuged to remove air bubbles prior to use.

Fluorescent dye (46955, Millipore Sigma) was aliquoted and then added to the bioinks for a final concentration of 0.01 mg of dye per mL of bioink. The dye was mixed during the last step prior to transferring to the printing syringe for each bioink, except for those which required centrifugation, in which case it was mixed during the final step prior to centrifugation.

### 2.2. Artifact printing

**Fig. 1.**
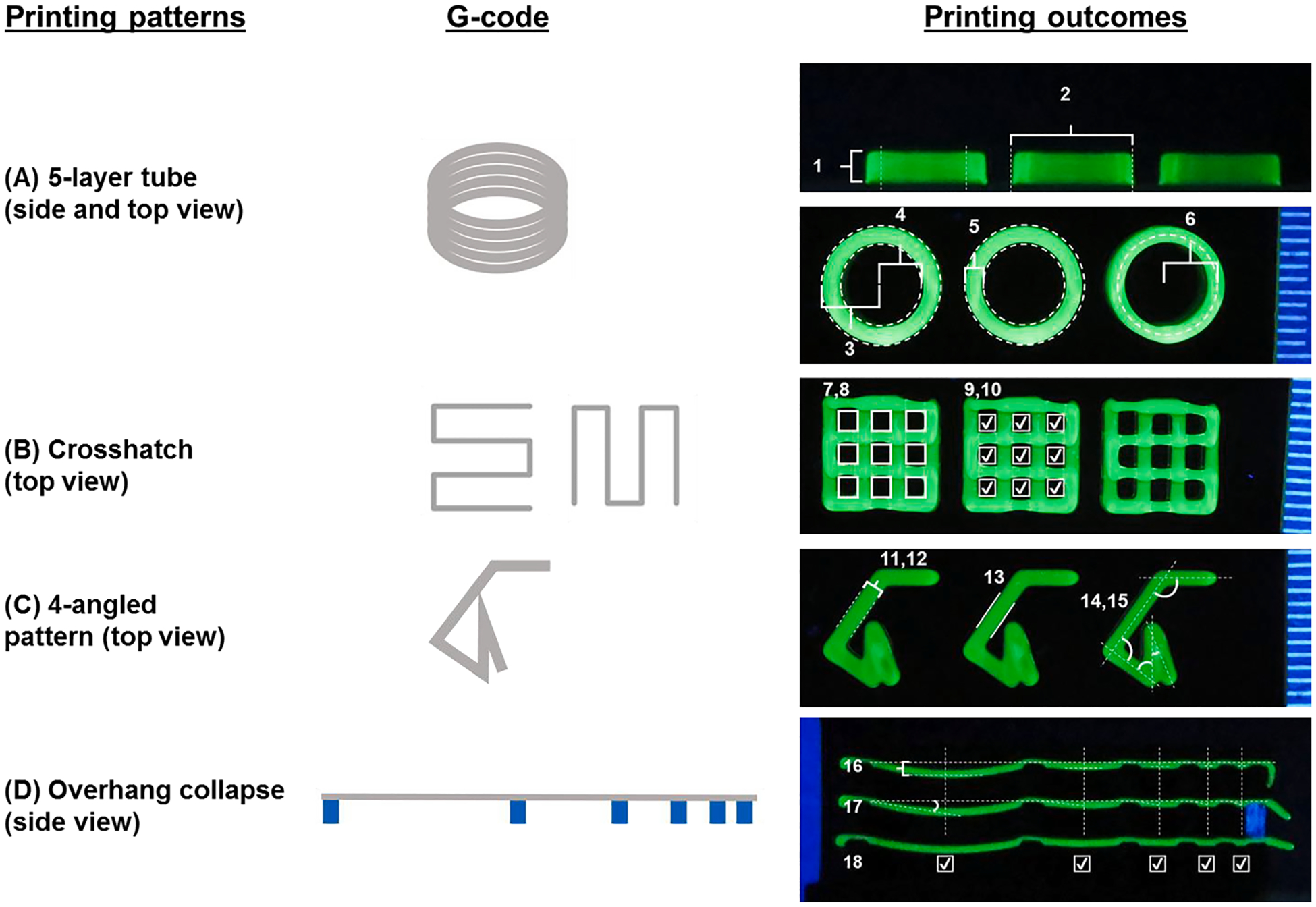
Bioink artifact set-up. Example images from the artifact labeled with each measurement made by the image analysis program: (A) 5-layer tube (side view and top view), (B) crosshatch, (C) 4-angled pattern, and (D) overhang. 1–18 correspond to the respective numbers in Table 1.

The artifact structures were printed onto a custom platform which itself was 3D printed on a MakerBot Replicator+ using polylactide (PLA) filament (MakerBot Industries, New York, NY). This platform contained pillars for the overhang collapse structures and a compartment that held three glass slides. The 5-layer tube, crosshatch, and 4-angled pattern were each printed on a separate glass slide held by the platform (Fig. 1).

The integrated tissue-organ printer (ITOP) and motion program, a custom-built platform previously developed by our laboratory, were used for this study [1]. The ITOP system is composed of an XYZ stage/controller and a dispensing module contained within an enclosed acrylic chamber. The dispensing module included a pneumatic pressure controller, plastic syringe, and cylindrical nozzle with an inner diameter of 330 μm. For all bioinks, the pressure was adjusted until a flowrate of 1.4 ± 0.05 mg/s was achieved, and this pressure was then used to print the artifact. Syringes were contained within a temperature-controlling sleeve (Musashi Engineering, Inc., Tokyo, Japan) with the temperature maintained at 21 ^◦^C unless otherwise noted. The ambient temperature was left at room temperature, which was maintained at approximately 20–22 ^◦^C. G-code for the printing pattern outlining and feedrate/print speed was generated in a custom, text-based software program. The printing pattern outlined the four structures, each in triplicate, at a feedrate of 150 mm/min, and a layer height of 0.42 mm. This G-code was then transferred to the motion controller program for printing the artifact (Supplementary Movie 1). After printing, the artifact was weighed to confirm that proper material deposition had occurred (400 ± 20 mg).

For the 5-layer tube, a circular printing path with a radius of 4 mm was used. As the outcomes are notable both in the horizontal and vertical planes, these structures were photographed from both above and from the side. Both height and width of these structures were measured. From above, external radius, internal radius, wall thickness, and radial accuracy were considered. A 2-layer crosshatch pattern, including 9 square-shaped pores arranged in 3 × 3 patterns, was included in the artifact. A line pitch of 2.67 mm, and therefore gross dimensions of 8 × 8 mm, was chosen based on expected filament resolution with the goal of individual pores which would be appropriately small without losing patency. First, whether each pore was filled or if any filaments were broken was observed. If patent and intact, *Pr*, as first described by Ouyang et al. [10], and pore area were measured for each pore. A 4-angled pattern was also incorporated into the artifact, including turns of 125^◦^, 90^◦^, 55^◦^, and 20^◦^. To maintain consistency with the crosshatch and 5-layer tubular structures, the 4-angled pattern was bound by an 8 × 8 mm square. In millimeter coordinates, the design started at one corner of this bounding square (0, 0) and moved to create the 125^◦^ turn at (4, 0), the 90^◦^ turn at (8, 5.7), the 55^◦^ turn at (4.7, 8), the 20^◦^ turn at (4.7, 4) and finished at (3.4, 7.8). The printed angle and angle error were measured for each of these turns. Additionally, the second line segment, the longest continuous line, was used for single-layer, single-filament analysis. The width, standard deviation of the width [18], and uniformity ratio [8] were taken from this segment. Lastly, the overhang collapse structure was printed on a plastic object with pillars that were 4 mm in height and 2 mm across [9]. To thoroughly test the capabilities of each bioink, a maximum gap of 16-mm was used between pillars, followed by gaps of 8-, 4-, 2-, and 1-mm. Spanning failure was defined as a break in the filament or contact with the lower substrate, corresponding to a deflection more significant than 4 mm. For each gap successfully crossed, the angle of deflection and deflection distance of the bioink was measured between the start of the gap and its midpoint.

### 2.3. Image capturing

After printing, the artifact was photographed using a PowerShot SX730 HS camera (Canon, Tokyo, Japan). To enhance the contrast between printed bioink and background, images were taken in a dark room with a black background. UV light filtered at 365 nm was shone on the structures using a flashlight (UV301D, LIGHTFE, Shenzhen, China). Additionally, a standard blue ruler was included in the same plane relative to the camera as the printed structures. Crosshatch, 4-angled pattern, and 5-layer tube structures were photographed from above at a distance of 5.5 cm, while overhang and 5-layer tube structures were photographed from the side at a distance of 90 cm and a zoom of approximately 1.2 m to acquire the appropriate perspective.

### 2.4. Image analysis measurement

Images were analyzed using a custom MatLab script (MathWorks, Natick, MA). Briefly, photographs were manually cropped to individual structures prior to importing. Fluorescent bioink was segmented using a mean shift algorithm. Objects and edges were then analyzed depending on the given structure. Areas were measured by a number of pixels (units: pixels^2^), and perimeters were calculated as the sum of the Euclidean distances between pixels (units: pixels). These measures were then later converted to mm using a conversion factor manually determined for each image using the ruler present in the same plane as the structures. For each measurement, the outcome of the PF bioink can be considered the ideal outcome except where the desired result is otherwise apparent.

5-Layer tube structures from the side demonstrate a rectangular, trapezoidal, or dome-like shape. The angled walls of the trapezoidal structures could disproportionately affect the height and width measures. Because of this, tube height was averaged over the middle portion, excluding the far left and far right sides by 20 pixels each. The width was taken as the maximum to capture the widest portion of the structure at its base. From above, internal and external radii were calculated by identifying the respective borders, measuring the areas within, and then calculating the average radius by assuming a circular shape. Wall thickness was then taken as the difference between the internal and external radii. Finally, radial accuracy was calculated as the average between the internal and external radii as a percentage of the designed radius, in this case, 4 mm.

For crosshatch structures, each pore was identified by its location. The number of pixels inside each pore was counted to measure the pore area. If a pore could not be detected, it was determined to be filled and assumed to have a pore area of zero. Broken pores could be detected if their area were the same as another pore or the area outside the structure. If a pore was broken, the area of that pore was no longer bounded along the broken edge. When this edge was located between two pores, the area from the adjacent pore was also included when the area of either pore was measured. When the broken edge was located on the outside border of the structure, the area from outside of the structure was also included. Repeated pore area results or an area equal to the outside of the structure was therefore indicative of a broken filament, except in the extremely rare case that the areas coincidentally had the same number of pixels. The perimeter for each pore was measured after using a Gaussian smoothing filter. This perimeter was then used in conjunction with the pore area to calculate the *Pr* value.

4-Angled pattern measurements were calculated by first isolating the individual straight segments based on their expected location within the image. A line of best fit was projected through each of the segments, which excluded the corners themselves, and the angle was then measured between the two projected lines, which were adjacent to each turn. Error for each angle was calculated by subtracting the designed angle from the measured angle to normalize for comparison across angles. Additional measures were taken from the most extended segment, which occurred between the 125^◦^ and 90^◦^ turns. First, the average width was taken by counting the distance between edges orthogonally to its best-fit line, and the standard deviation was also calculated for these measurements. The perimeter for both edges of this segment was measured after first applying a Gaussian smoothing filter. These two perimeters were averaged and then normalized by the straight-line length of the segment along the best-fit line.

For overhang structures, the supporting pillars were first identified by detecting the locations where the bioink conformed to their surface. Next, midpoints between each pillar were also found. The center location of the filament was then identified for each pillar edge and midpoint. The deflection was measured as the vertical distance between these two locations for each gap. Angle was then calculated relative to the horizontal using this vertical and horizontal distance. If the deflection distance was more significant than 3.88 mm or if any discontinuities were detected between pillars, the filament was considered to have failed at that gap, and deflection distance and angle were not measured.

### 2.5. Rheological measurement

All rheological tests were carried out on a Discovery Hybrid Rheometer-2 (TA Instruments, Wilmington, DE) using a gap of 100 μm and a 40 mm cone-plate geometry with a 1^◦^ angle. Frequency sweeps, strain sweeps, and recovery tests were conducted in triplicate to determine each bioink’s shear-thinning, viscoelastic, yielding, and recovery behaviors. Frequency sweeps were conducted at a strain of 0.2%. Frequencies ranged from 0.01 to 100 Hz using a logarithmic sweep with 10 points per decade. The Power-law equation (1) was fitted to shear rate vs. complex viscosity data:

(1) $$\eta \;=\;K{\dot{\gamma }}^{n-1}$$

Where is the shear rate, *η* is the viscosity as measured by the rheometer, and *K* and *n* are estimated constants known as the consistency index and flow index, respectively.

For bioinks that do not fit the power-law model, the Herschel-Bulkley model was used to fit shear stress vs shear rate. The Herschel-Bulkley equation (2) is expressed as:

(2) $$\tau \;=\;\tau_{0}\;+\;K{\dot{\gamma }}^{n}$$

Where *τ* is the shear stress, *τ*_0_ is the yield stress, $\dot{\gamma }$ is the shear rate, and *K* and *n* are again estimated constants known as the consistency index and flow index, respectively. The consistency index (*K*) and flow index (*n*) of each bioink were used for further analysis [12,14,19,20].

Strain sweeps were conducted at a frequency of 1 Hz. Applied strain ranged from 0.1% to 1000% using a logarithmic sweep with 10 points per decade. Stress sweeps were also performed on the bioinks for cases during which a strain of 0.1% would put it past its yield point. For these samples, a stress of 0.1 to 100 Pa was applied using a logarithmic sweep with 10 points per decade. Storage modulus (G’) and loss modulus (G”) were averaged at low strains within each bioink’s linear viscoelastic region. Loss tangent (tan(δ)) was calculated as the ratio G”/G’ from these values. For bioinks that exhibited yielding behavior (G’ > G”), initiation of flow was defined as the crossover point between G’ and G” and the yield stress was determined by the stress which occurred at that point. For each bioink, G’, G”, tan(δ), and, if applicable, yield stress was used for further analysis.

Recovery tests were conducted in three phases to model the extrusion process. The first phase resembles conditions in the syringe prior to extrusion, and a shear rate of 1/s is applied to the bioink for 30 sec. The second phase resembles conditions in the nozzle during extrusion, and a shear rate of 400/s is applied to the bioink for 10 sec. The third and final phase resembles conditions on the printing substrate after extrusion, and a shear rate of 1/s is applied to the bioink for 120 sec. Several measures were taken from each test. Initial viscosity was averaged from the final 5 sec of the first phase. Viscosity was measured during the third phase at 3 and 80 sec. Because all bioinks remain in the general range of their original viscosities, it can be useful to evaluate the recovered viscosity as a percentage of the initial viscosity. The percentage of initial viscosity recovered at 3 and 80 sec was used for further analysis.

### 2.6. Statistical analysis

Each experiment was carried out in triplicate. Statistical analyses were conducted via JMP 13 software (SAS Institute Inc., Cary, NC) with an α level of 0.05. Unless otherwise noted, all comparisons were made via One-way analysis of variance (ANOVA), with *post-hoc* analyses conducted using *Tukey’s* Honestly Significant Difference between means for each combination.

## 3. Results

### 3.1. Quantitative printing outcomes measured by bioink artifact

For quantitative printing outcome measurement, an extrusion bioink-specific artifact was developed to improve the quantification, comprehensiveness, and standardization of printability assessment (Fig. 1 and Table 1). In the design criteria and considerations, the artifact’s structures included those commonly used in the target applications and sufficient to demonstrate the maximum capabilities and limitations of a printing system and material. In this study, seven bioinks were chosen for this study to represent a wide range of potential outcomes (Supplementary Table 1).

#### 3.1.1. 5-Layer tube

**Fig. 2.**
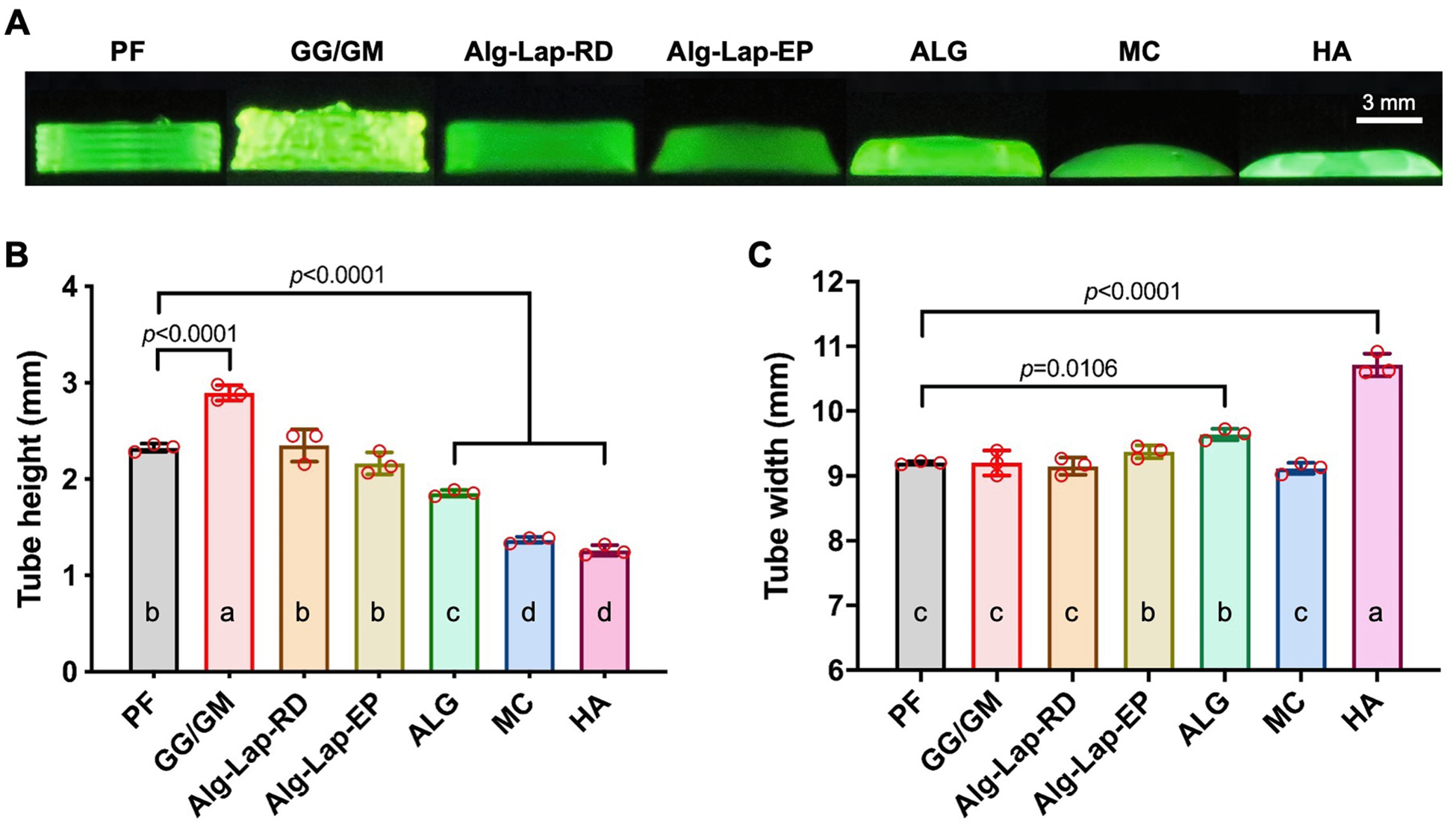
Results from the side view of 5-layer tube structures for each bioink, including (A) photographs, (B) tube height, and (C) maximum tube width. a,b,c,d denotes statistical significance (*p* < 0.05). Levels not connected by the same letter are considered significantly different.

Tube height and tube width were measured for each bioink (Fig. 2A). Generally, a higher tube height is more desirable, up to the designed height of 2.1 mm, demonstrating a bioink’s ability to stack multiple layers. GG/GM resulted in the highest structure with an average height of 2.90 ± 0.08 mm, 0.80 mm taller than the designed height. This is possible due to the die swell of the filaments and the bioink’s resistance to deformation by the nozzle after extrusion. Conversely, the layers of PF, Alg-Lap-RD, and Alg-Lap-EP showed a more flattened geometry and therefore did not rise as much above the tip of the nozzle (2.33 ± 0.04, 2.35 ± 0.17, and 2.16 ± 0.12 mm, respectively). ALG showed a lesser ability to stack layers and was unable to reach the designed height (1.85 ± 0.03 mm). The bioinks with the least shape fidelity, MC (1.37 ± 0.03 mm) and HA (1.26 ± 0.06), demonstrated the lowest values for measured height (Fig. 2B). Tube width is another measure of multi-layer shape fidelity, with lower values indicating less collapse. However, it does not capture collapse in the inward direction. Tube width showed less variation between bioinks than tube height. HA was much wider than all other bioinks, with an average width of 10.71 ± 0.18 mm. Structures printed with ALG and Alg-Lap-EP also exhibited a slightly wider base (9.64 ± 0.09 and 9.37 ± 0.10 mm, respectively), statistically different from all other bioinks. No statistically significant differences were detected between all other bioinks (*p* > 0.197 for all combinations) (Fig. 2C).

**Fig. 3.**
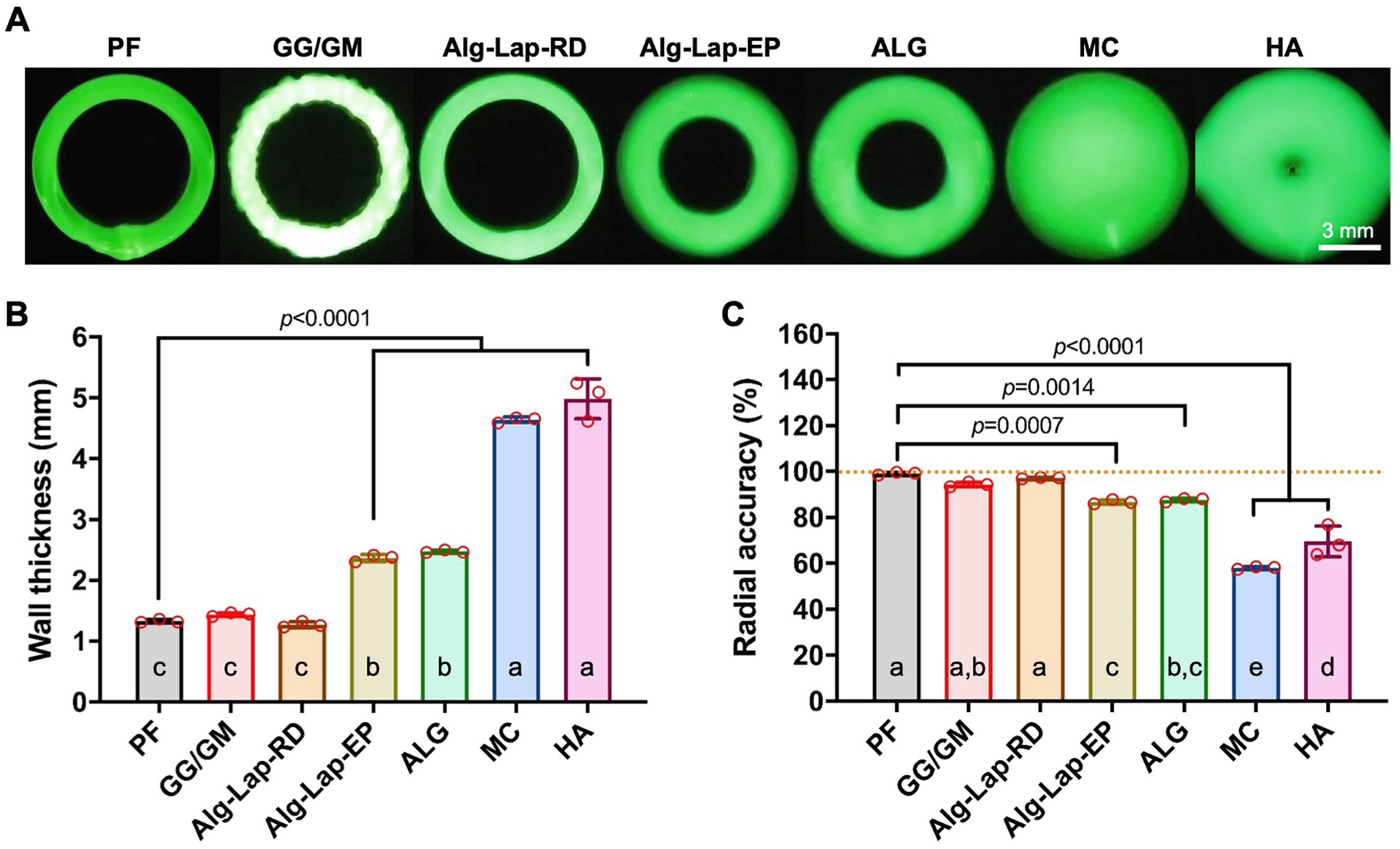
Results from the top view of 5-layer tube structures for each bioink, including (A) photographs, (B) tube height, and (C) maximum tube width. a,b,c,d,e denotes statistical significance (*p* < 0.05). Levels not connected by the same letter are considered significantly different.

External radius, internal radius, wall thickness, and radial accuracy were measured for each bioink (Fig. 3A). External radius is another measure of outward tube collapse, except captured from above and taken as an average rather than a cross-section. HA had the greatest external radius (5.28 ± 0.15 mm), followed by ALG. ALG (4.74 ± 0.02 mm) was only shown to be greater than Alg-Lap-RD (4.52 ± 0.02 mm) and GG/GM (4.49 ± 0.04 mm). No differences were detected between all other bioinks (*p* > 0.092 for all combinations). The differences between bioinks were considerably greater for the measure of internal radius, which captured the inward collapse of the tubular structure. The high shape fidelity bioinks, PF (3.30 ± 0.01 mm), Alg-Lap-RD (3.25 ± 0.04 mm), and GG/GM (3.05 ± 0.04 mm), showed the highest internal radii. As shape fidelity decreased, these were followed by Alg-Lap-EP (2.29 ± 0.06 mm) and ALG (2.27 ± 0.04 mm). Lastly, tubes printed with HA and MC were all fully or nearly collapsed with internal radii of 0.29 ± 0.29 mm and 0.00 ± 0.00 mm, respectively. Wall thickness is a function of the internal and external radius and therefore showed similar trends. HA (4.98 ± 0.33 mm) and MC (4.64 ± 0.05 mm) showed the highest thicknesses, followed by ALG (2.47 ± 0.02 mm) and Alg-Lap-EP (2.36 ± 0.06 mm) with improved shape fidelity. GG/GM (1.44 ± 0.03 mm), PF (1.33 ± 0.03 mm), and Alg-Lap-RD (1.27 ± 0.05 mm) were able to print tubular structures with the thinnest walls, which is generally more desirable (Fig. 3B). Finally, not only was the magnitude of these dimensions considered, but the accuracy relative to the 4 mm designed printing path was taken as well. PF (99.13 ± 0.58%), Alg-Lap-RD (97.18 ± 0.28%), and GG/GM (94.35 ± 0.99%) all showed excellent radial accuracy, nearly 100% of the designed path. Radial accuracy for other bioinks was decreased, primarily by their reduced internal radius, reaching values as low as 58.01 ± 0.56% for the completely collapsed MC (Fig. 3C). Values for all 5-layer tube measurements are listed in Supplementary Table 2.

#### 3.1.2. Crosshatch

**Fig. 4.**
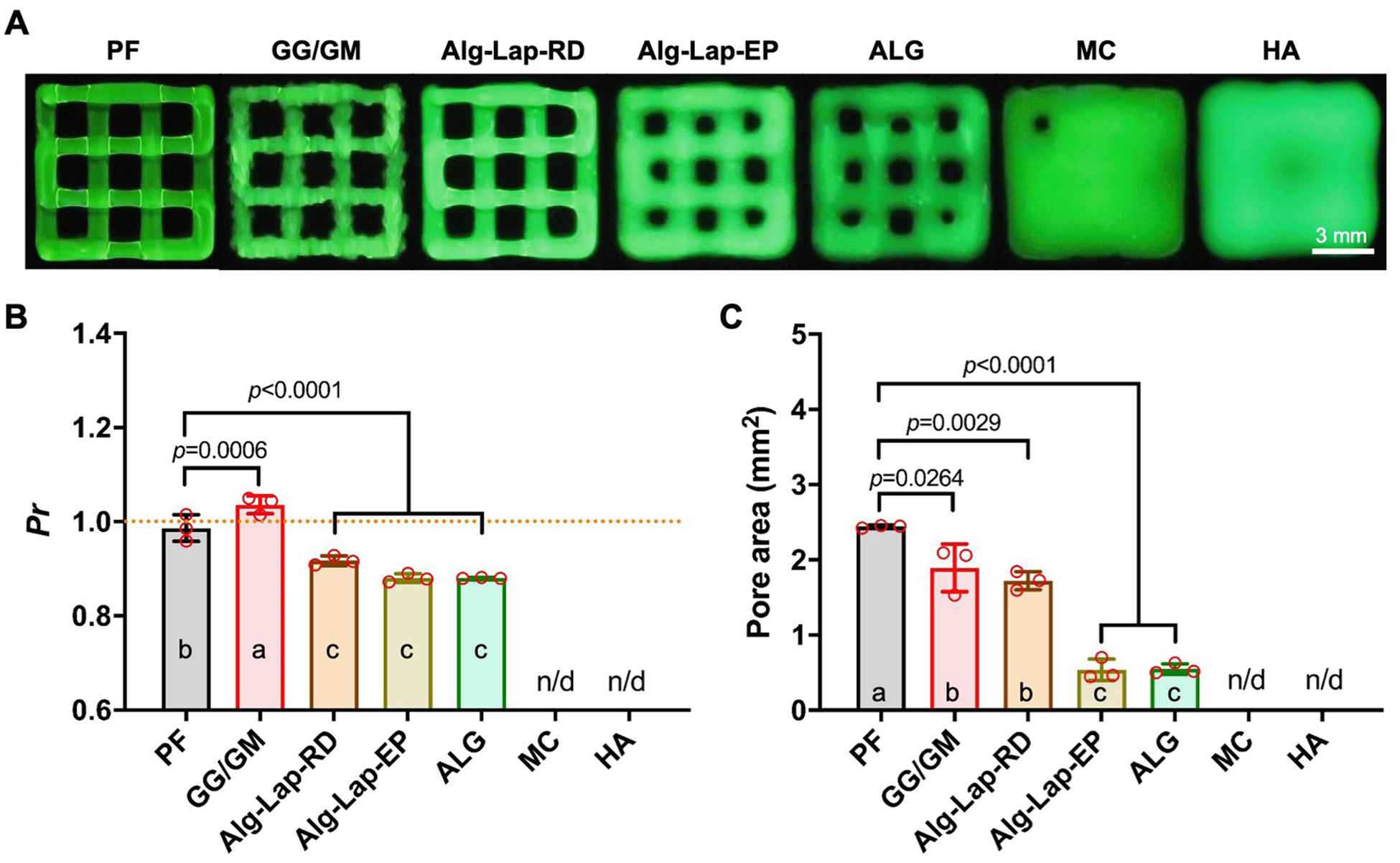
Results from crosshatch structures for each bioink, including (A) photographs, (B) *Pr*, and (C) pore area. a,b,c denotes statistical significance (*p* < 0.05). Levels not connected by the same letter are considered significantly different.

Pore areas and *Pr* values were measured for each pore for each bioink. Due to insufficient shape fidelity for MC and HA, all pores were completely filled in for each testing replicate and, therefore could not be measured (Fig. 4A). For comparisons between bioinks, all pores within a given structure were first averaged together. The non-uniform filaments of GG/GM showed a slightly raised *Pr* value of 1.04 ± 0.02, and PF resulted in a near-perfect *Pr* value of 0.99 ± 0.03. The more circular pores of Alg-Lap-RD (0.92 ± 0.01), ALG (0.88 ± 0.01), and Alg-Lap-EP (0.88 ± 0.01) all showed similarly low *Pr* values (Fig. 4B). In general, larger pore areas are more desirable for bioink applications, notwith-standing study-specific design considerations. The largest pore area was measured for PF (2.44 ± 0.02 mm^2^) followed by GG/GM and Alg-Lap-RD (1.89 ± 0.32 and 1.72 ± 0.12 mm^2^, respectively). Alg-Lap-EP and ALG (0.54 ± 0.14 and 0.55 ± 0.07 mm^2^, respectively) showed lower pore areas than the other bioinks and no difference between each other (Fig. 4C). Values for all crosshatch measurements are listed in Supplementary Table 2.

#### 3.1.3. 4-angled pattern

**Fig. 5.**
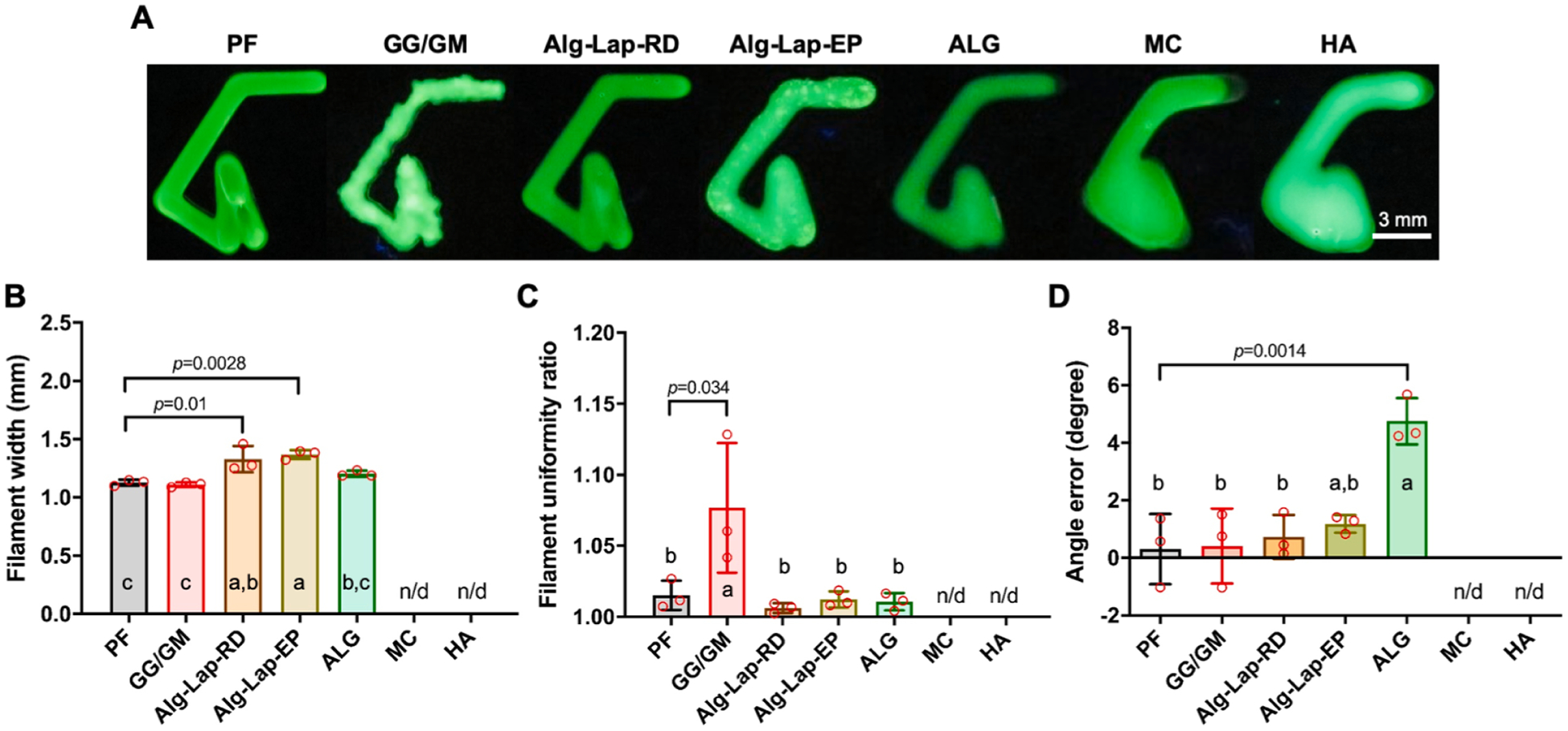
Results from 4-angled patterns for each bioink, including (A) photographs, (B) filament width, (C) uniformity ratio, and (D) turn angle error. a,b,c denotes statistical significance (*p* < 0.05). Levels not connected by the same letter are considered significantly different.

Filament width, filament width standard deviation, uniformity ratio, angles, and angle errors were measured for each bioink except MC and HA, which could not be measured due to insufficient shape fidelity (Fig. 5A). Filament width was lowest among GG/GM (1.11 ± 0.02 mm), PF (1.13 ± 0.03 mm), and ALG (1.20 ± 0.03 mm) bioinks. Of note, Alg-Lap-EP (1.37 ± 0.04 mm) and Alg-Lap-RD (1.33 ± 0.11 mm) showed the highest filament width (Fig. 5B). Unlike similar measurements from the crosshatch and 5-layer tube structures, these values did not correspond to the bioink’s shape fidelity. The standard deviation of the filament width is a measure of filament uniformity, with lower standard deviations being more desirable. This measure was elevated in the non-uniform GG/GM (*p* < 0.009 compared to all other bioinks) with a value of 0.114 mm. No differences were detected between the uniform bioinks (*p* > 0.305 for all other combinations), with values ranging from 0.019 to 0.050 mm. Similarly, a uniformity ratio of 1 represents greater uniformity and therefore is more desirable. The uniformity ratio was increased in GG/GM (*p* < 0.034 compared to all other bioinks) with a value of 1.077 ± 0.046. No differences in uniformity ratio were detected between other bioinks (p > 0.984 for all other combinations) with values ranging from 1.006 ± 0.003 to 1.015 ± 0.010 (Fig. 5C). The angle of each turn was also quantified. These turns were designed to have successively decreasing angles of, 90^◦^, 55^◦^, and 20^◦^. Angle error was calculated at each turn to allow for comparisons across different angles. GG/GM (0.54 ± 2.74^◦^), PF (0.30 ± 1.92^◦^), and Alg-Lap-RD (0.72 ± 0.98^◦^) bioinks showed excellent turn-angle errors, <2^◦^ for all angles. Alg-Lap-EP showed a slightly larger turn angle error (1.18 ± 3.64^◦^). Turn angle error was significantly higher for ALG (4.75 ± 5.27^◦^) (Fig. 5D). Values for all 4-angled pattern measurements are listed in Supplementary Table 3.

#### 3.1.4. Overhang collapse

**Fig. 6.**
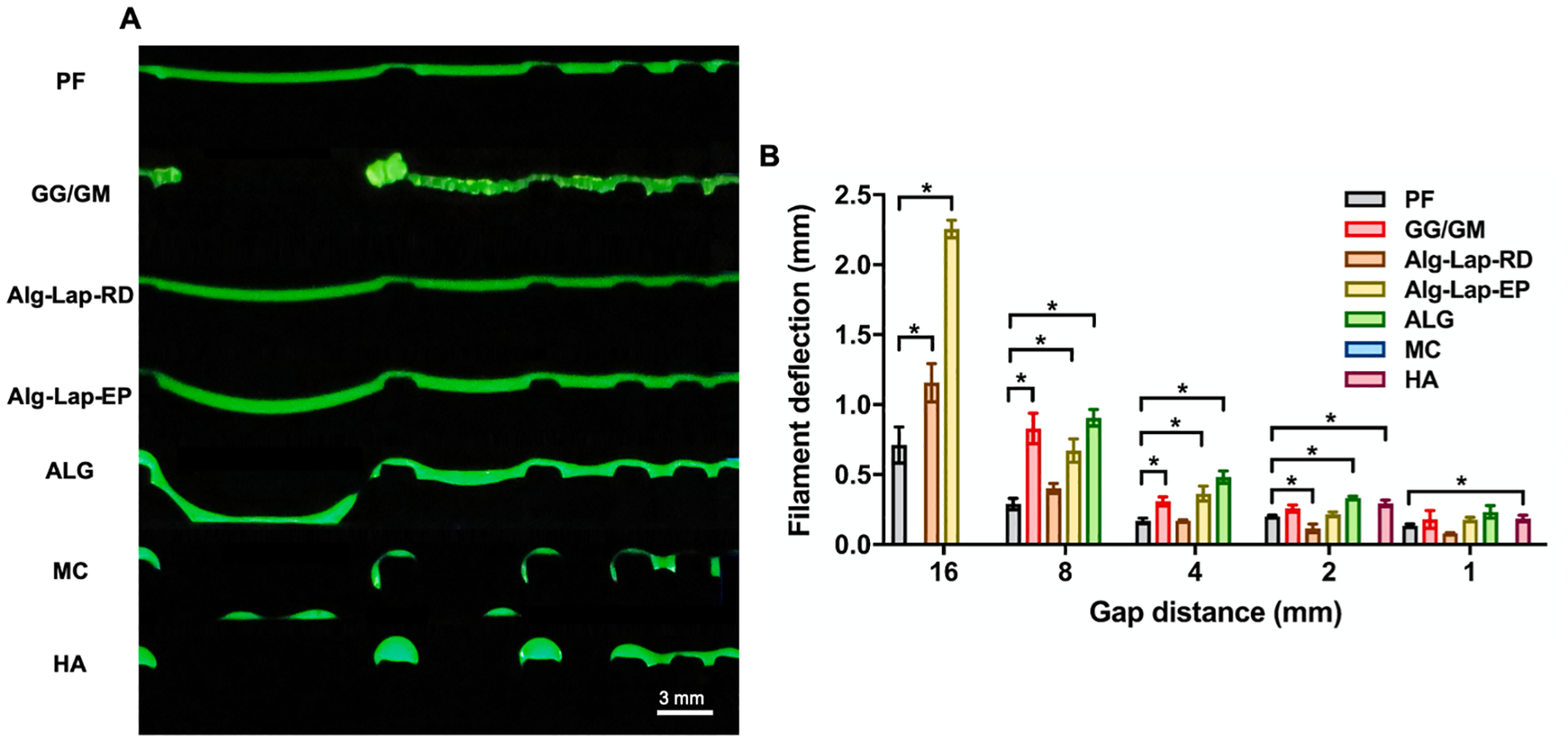
Results from overhang structures for each bioink, including (A) photographs and (B) filament deflection distance at each gap length. * denotes statistical significance (*p* < 0.05).

Whether the filament successfully spanned each gap was determined for each bioink. If successful, deflection distance and angle were also measured. MC was detected as unable to span any of the gaps successfully while HA filaments only traversed the 1-mm and 2-mm gaps without breaking. ALG slightly improved upon these two, successfully crossing all gaps except the 16-mm. For GG/GM, 16-mm gap, one testing replicate successfully spanned the gap while the other two failed. However, testing replicates were consistent for all other bioinks and gap lengths. Three bioinks, PF, Alg-Lap-RD, and Alg-Lap-EP, were able to span all gaps (Fig. 6A). When comparing between bioinks, the results align well with visual expectations. Low deflection distances at 16-mm, 8-mm, and 4-mm gaps were seen for PF and Alg-Lap-RD. This relationship continued at 2-mm and 1-mm, although the effect was slightly diminished. Differences between the two were detected at the 16-mm gap with a deflection of 0.71 ± 0.13 mm for PF and 1.15 ± 0.14 mm for Alg-Lap-RD (*p* = 0.012). Notably, despite being dissimilar by other measures, GG/GM performed similarly to Alg-Lap-EP at all gaps. Except at 1-mm, Alg-Lap-EP also slightly outperformed ALG at all gaps, despite producing similar results in other structures. Deflection distances for HA were similar to all other bioinks except PF and Alg-Lap-RD, although it only successfully spanned the 2-mm (0.30 ± 0.02 mm) and 1-mm gaps (0.19 ± 0.02 mm) (Fig. 6B). The deflection angle for each bioink was also measured. However, since this value can be directly calculated from deflection distance and gap length, relationships between bioinks remain unchanged whether the analysis is conducted from the deflection angle or deflection distance. Values for all overhang measurements are listed in Supplementary Table 4.

To characterize the overall performance of each individual bioink, the printability outcomes were visually graded as either: Good, Intermediate, Poor, or non-detectable (n/d). For each metric, the printing outcome of each bioink was compared to the CAD-based theoretical outcome. If the printing outcome visually looked the same as the theoretical outcome, it was considered “+++ Good.” If it appeared similar but with some slightly noticeable differences, it was considered “++ Intermediate.” And if the outcome was not recognizable as the theoretical outcome, it was graded “+ Poor”, and, lastly, outcomes that could not be measured were graded as “n/d: non-detectable.” These results were compiled into Table 2, highlighting the decision to use PF as the standard bioink as it exhibits “Good” printability across all measured metrics.

### 3.2. Rheological properties of bioinks

**Fig. 7.**
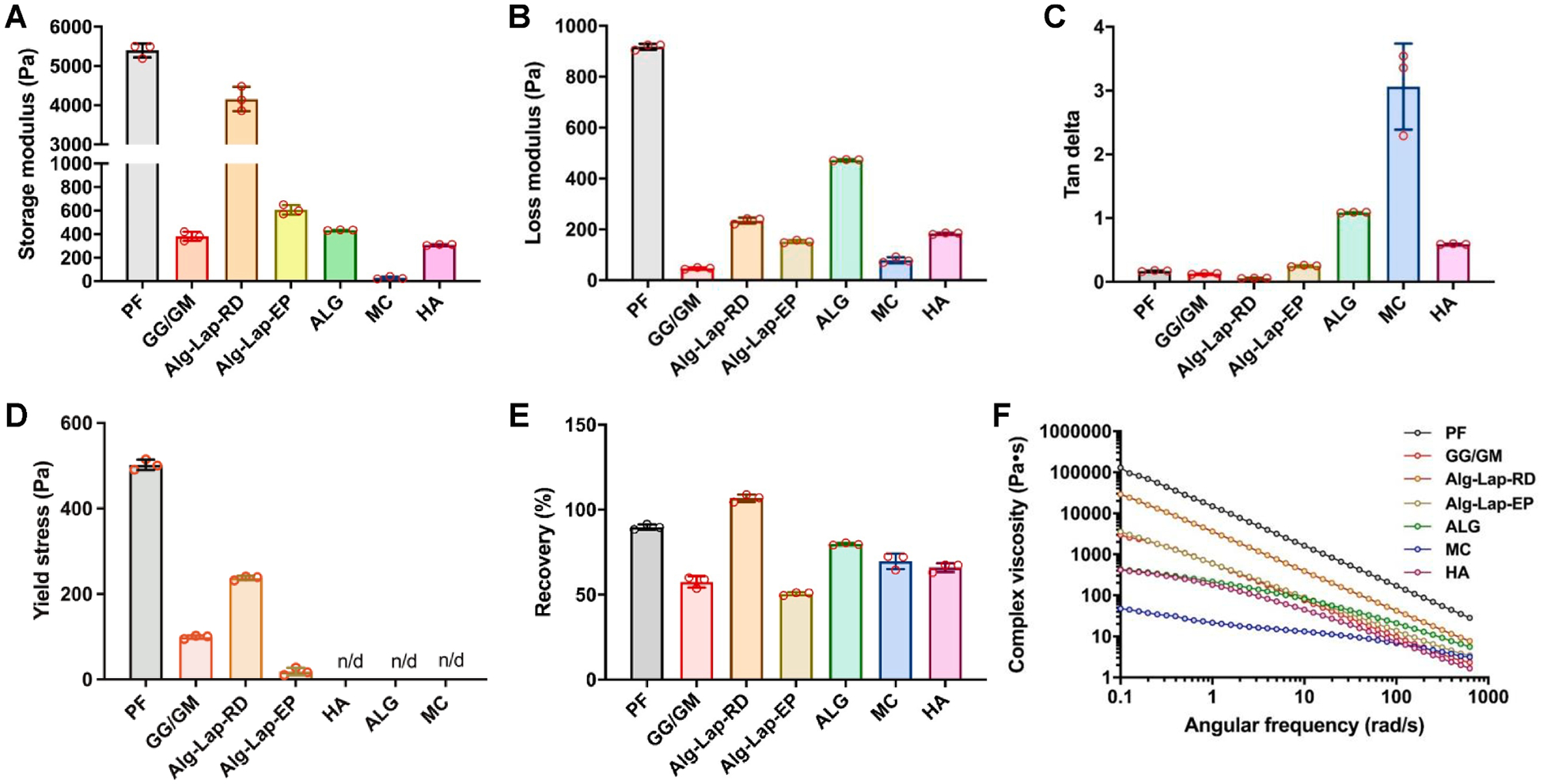
Results from rheological testing of different bioinks. Strain sweeps were used to determine (A) storage modulus, (B) loss modulus, (C) tan delta, and (D) yield stress. (E) Recovery tests were conducted to determine the recovered viscosity after 3 sec as a percentage of the initial viscosity. (F) Frequency sweeps were used to determine the bioinks’ shear-thinning abilities.

Strain sweeps revealed a wide range of behaviors between the different bioinks. PF and Alg-Lap-RD showed by far the largest storage moduli (5395 ± 177 Pa and 4161 ± 310 Pa, respectively). Compared to these two, GG/GM showed similar shape fidelity by several measures but had a lower storage modulus (382 ± 38 Pa) than even Alg-Lap-EP (608 ± 41 Pa) and ALG (434 ± 4 Pa). A low storage modulus was also found for HA (310 ± 5.0 Pa) and was near zero for MC (28 ± 11.1 Pa) (Fig. 7A). Bioinks varied to a lesser degree in their loss moduli. Compared to other bioinks, PF still had a much higher loss modulus (918 ± 12 Pa), but this difference was much less extreme than for storage modulus. On the other hand, Alg-Lap-RD showed a loss modulus (235 ± 11 Pa) which was more in line with that of the other bioinks such as HA (184 ± 3 Pa) and Alg-Lap-EP (152 ± 5 Pa). Instead, ALG showed the second-highest loss modulus (473 ± 3 Pa). Both MC (80 ± 11 Pa) and GG/GM (47 ± 4 Pa) had notably low loss moduli (Fig. 7B). Tan(δ) represents the ratio between storage and loss modulus. Alg-Lap-RD (0.06 ± 0.002), GG/GM (0.12 ± 0.004), PF (0.17 ± 0.007), and Alg-Lap-EP (0.25 ± 0.008) each showed very low tan(δ). Tan(δ) increased dramatically for other bioinks with HA (0.59 ± 0.001), ALG (1.09 ± 0.006), and MC (3.06 ± 0.678) each marking notable jumps (Fig. 7C).

The yield stress was not identified for MC or ALG since both had higher loss moduli than storage moduli. Both Alg-Lap-EP and GG/GM yielded at low stresses of only 18 ± 9 Pa and 99 ± 4 Pa, respectively. The yield stress of Alg-Lap-RD was more moderate at 327 ± 5 Pa. Notably, HA showed even higher yield stress (536 ± 2 Pa) than PF (502 ± 12 Pa), despite being dissimilar by all other printability and rheological measures (Fig. 7D).

Recovery tests were used to model the extrusion process. All bioinks showed a rapid decrease in viscosity in response to the increased shear rate, followed by some degree of rapid recovery after its removal. While the speed and magnitude of these recoveries varied from bioink to bioink, the recovered viscosities largely were dependent on the initial viscosity. From this, PF and Alg-Lap-RD had the most complete recovery, both in terms of speed and magnitude, with recoveries greater than 90% by 3 sec (Fig. 7E). Slow recoveries were seen for GG/GM, MC, and HA, which all increased in viscosity dramatically between 3 and 80 s from 58% to 76%, 70% to 93%, and 66% to 88%, respectively. Both ALG and Alg-Lap-EP showed rapid recoveries with little change between 3 and 80 sec. However, the magnitudes of these recoveries were moderate for ALG (80% to 87%) and poor for Alg-Lap-EP (51% to 62%).

Finally, all bioinks showed shear-thinning behavior during frequency sweeps. Viscosity was markedly higher in PF, although Alg-Lap-EP also showed a much higher viscosity than the other bioinks with *K* constants of 11,437 and 2755, respectively (Fig. 7F). These high viscosities were partially offset by extremely low flow constants (0.196 and 0.197, respectively), indicating they were also the most shear-thinning of the bioinks. High shear-thinning abilities were also seen for GG/GM (*K* = 98.7, *n* = 0.27). Shear-thinning decreased with 3% HA (*K* = 558, *n* = 0.78), Alg-Lap-RD (*K* = 28.3, *n* = 0.80), and again with ALG (*K* = 8.5, *n* = 0.70). MC showed slightly high viscosity and only slight shear thinning behavior with a flow constant (*n*) of 0.82 and consistency index (*K*) of 1456. Values for all rheology measurements are listed in Supplementary Table 5.

### 3.3. Relationship between rheological properties and printing outcomes

To relate these rheological results to the artifact outcomes, each rheological measure was plotted against each printing outcome and a linear regression analysis was conducted. This resulted in 314 combinations for analysis. While reporting on each of these combinations would be neither feasible nor productive, some observable patterns were identified (Supplementary Fig. 1 and Supplementary Fig. 2). Both high printability bioinks (PF and Alg-Lap-RD) had particularly high storage moduli, approximately 10 times higher than that of the other bioinks. However, GG/GM and Alg-Lap-EP both had reasonably good shape fidelity by several measures, matching or coming close to the high printability bioinks. Storage modulus alone would have underestimated GG/GM in particular, whose storage modulus was lower than ALG and not far above HA. Loss modulus appeared to have no clear relationship with printing outcomes when considered by itself for these bioinks. Loss modulus was elevated in PF, middle of the group for Alg-Lap-RD, and near-zero for GG/GM. A low tan(δ) could be considered a good predictor for high shape fidelity as it was particularly low for Alg-Lap-RD, GG/GM, PF, and Alg-Lap-EP. Although, when comparing between these bioinks, tan(δ) would have underestimated PF, whose tan(δ) was significantly higher than both GG/GM and Alg-Lap-RD. Tan(δ) also would have underestimated ALG, which had a much higher tan(δ) than Alg-Lap-EP and HA despite performing similarly for many artifact measures to Alg-Lap-EP and outperforming HA on all artifact measures.

The yield stress was similarly inconclusive, generally tracking well with artifact outcomes with a few stark exceptions. Although it had mediocre printing outcomes, ALG could not be measured due to its loss modulus being greater than its storage modulus. Similarly, the yield stress of HA was not measurable even at the lowest shear rates and shear stresses applied to the sample, which indicated it was flowing even without any shear stress. Lastly, Alg-Lap-RD had vastly different rheological properties, especially considering their yield stress, even though the two bioinks could be differentiated only by their overhang deflection at the 16-mm gap. Measures of bioink recovery also trended well with artifact performance. Alg-Lap-RD and PF had the best recovery capabilities, followed by ALG, then MC, and then HA, tracking with their ranking by most printability measures. However, GG/GM and Alg-Lap-EP recovered the lowest percentages of their viscosities, worse than even HA, in contradiction to various artifact measures which showed them superior to HA, MC, and ALG.

**Fig. 8.**
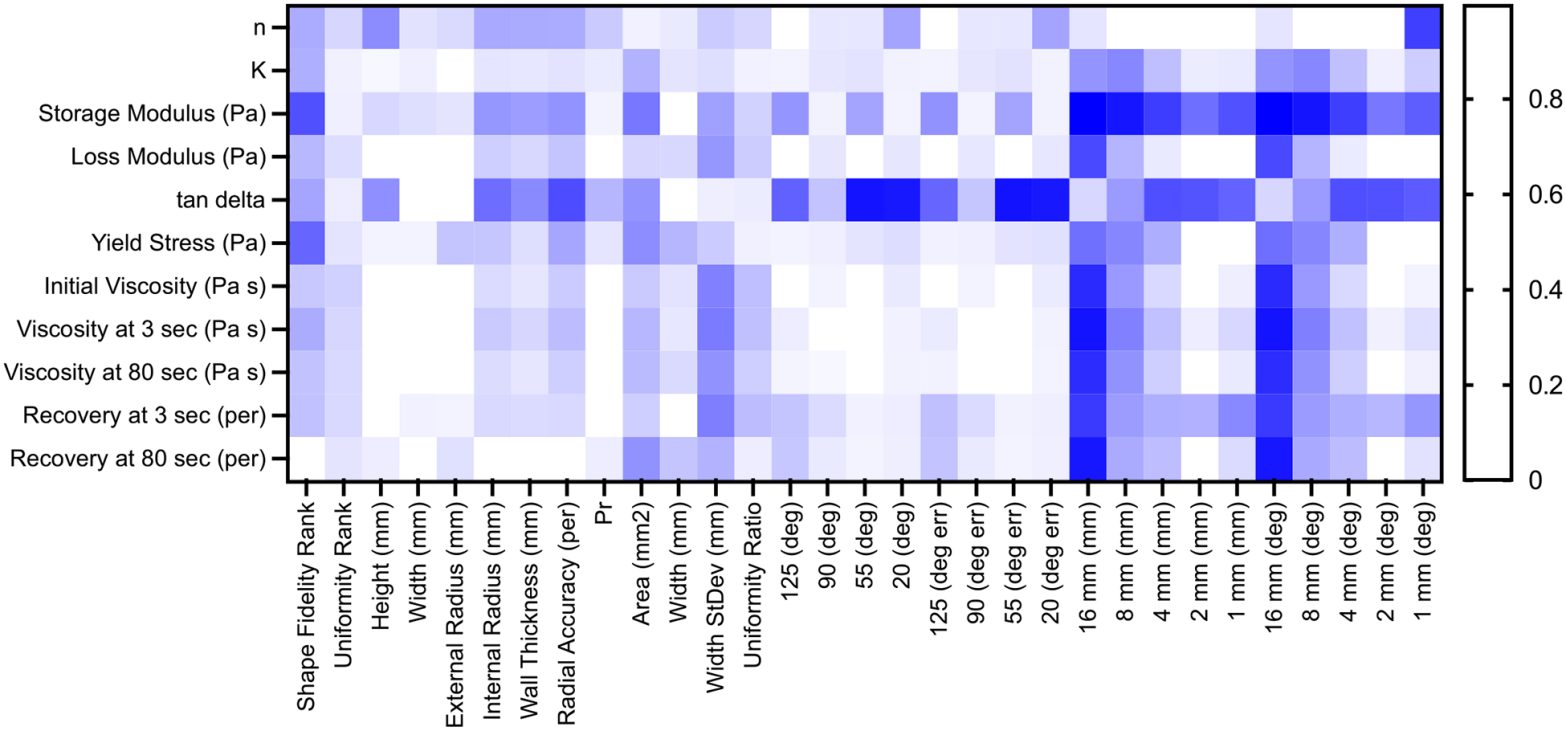
Linear regression analysis between rheological parameters and bioink artifact printing outcomes.

The linear regression analysis was performed on the printability measurements of each bioink as well as the rheological measurements and compiled into a heatmap to easily distinguish trends between measures (Fig. 8).

## 4. Discussion

Developing a comprehensive printability artifact holds great potential as a standardized tool for the bioink development process. In designing the artifact, we identified various structures commonly used in bioprinting applications, specifically focusing on structures previously utilized for evaluating printability [4]. Each structure serves a unique purpose in assessing different aspects of printability.

Arcs, representing distinct paths based on the desired printing geometries, were incorporated into the artifact design. The previously established 5-layer tubular structure allows for the analysis of these arc paths [8]. The symmetrical geometry of this structure allows for evaluating the impact of stacking multiple layers, as any structural deformations occur uniformly in all directions. The inclusion of a crosshatch structure is vital, as it mimics the common internal pattern observed in bioprinting. These porous regions play a critical role in facilitating nutrient diffusion to cells located in the deeper regions. Therefore, the size, shape, and patency of these pores become significant outcomes during the printing process. Sharp turns pose a challenge in the creation of bioprinted constructs [21]. The resolution of corners also influences the success of achieving the desired overall geometry. To address these aspects, the 4-angled pattern was integrated into the artifact, enabling the assessment of printing outcomes related to individual turns. Additionally, unsupported filaments represent another important feature encountered in bioprinted constructs. These filaments lack support from another filament directly beneath them, especially when creating vertical pores. To evaluate such printing outcomes, overhang structures were incorporated into the artifact design. By incorporating these patterns into the artifact, we aimed to capture the overall printability of the bioink comprehensively without the need for repetitive outcome measurements. This comprehensive approach allows for a more thorough evaluation of bioink performance, ultimately contributing to the advancement of bioink development for bioprinting applications.

The comprehensive printability artifact offers a valuable tool for thoroughly characterizing bioink printing outcomes, addressing the limitations of individual printability tests. Relying on a single measure, such as the 5-layer tube, can lead researchers to mistakenly conclude that Alg-Lap-EP and GG/GM exhibit similar and overall good printability, overlooking Alg-Lap-EP’s poor performance across various metrics. Similarly, relying solely on the overhang collapse test may falsely suggest that only PF and Alg-Lap-RD possess good printability, neglecting the underlying complexity. These findings can be validated through comparisons with other studies that have employed single measures from the artifact, including uniformity ratio, tube height, overhang collapse, and *Pr* value. Such comparisons consistently reveal discernible distinctions between “good” and “bad” outcomes within each artifact pattern. Moreover, linear regression analysis indicates the minimal correlation between printing outcomes obtained from different patterns, highlighting their distinct and unique information contributions. Considering the results obtained from multiple patterns within the artifact, a comprehensive understanding of bioink printability can be attained. This approach empowers researchers with valuable insights into the nuanced and multifaceted performance of diverse bioinks during the printing process, facilitating more informed decision-making in bioink selection and optimization.

To explore the link between rheological properties and printing outcomes, we conducted a study using a diverse set of seven hydrogel-based bioinks with varying expected printability. To establish a bench-mark for high printability, PF (Poloxamer 407), a bioink known for its exceptional printability in previous studies [9,22,23], was utilized. Similar results have been seen with Alg-Lap-RD, another bioink with demonstrated favorable printability [11,24], which served as a secondary high printability comparator. Both PF and Alg-Lap-RD exist in a gelled state and exhibit paste-like behavior. In contrast, two bioinks with poor shape fidelity, 8% MC and 3% HA, were included in the study. Furthermore, three bioinks with varying degrees of printability between the high and low groups were included: Alg-Lap-EP, ALG, and GG/GM. These intermediate bioinks were selected to ensure that the artifact could effectively differentiate printability outcomes that might not be readily discernible through visual observation. The bioprinting flowrate and feedrate employed in this study were determined based on their speed ratio, which has previously been demonstrated to yield successful printing outcomes for the crosshatch and 5-layer tubular structures in various bioinks [25]. Using these predetermined parameters, the study aimed to ensure consistent and comparable assessments across different bioinks and printing structures.

The rheological measurements agreed well with those from previous studies where similar data is available, including HA [26], Alg-Lap-RD [11,27], ALG [8], and PF [9]. The high printability bioinks (PF and Alg-Lap-RD) demonstrated a high consistency index (*K* constant), low flow index (*n* constant), high storage modulus, low tan(δ), high yield stress, and a viscosity recovery which was both rapid and nearly complete relative to the other bioinks. During the comparison of rheological properties with printing outcomes, several noteworthy observations were derived from the linear regression analyses. The storage modulus strongly correlated with most of the overhang testing outcomes. This finding aligns logically with the understanding that materials with higher stiffness are more likely to span a gap without significant deflection effectively. Furthermore, the outcomes related to the 16-mm gap demonstrated a notable correlation with various viscosity measurements. This observation is consistent with the expectation that materials with lower viscosity would face challenges in successfully bridging larger gaps.

Surprisingly, the yield stress, often regarded as a predictive measure for bioink extrusion, exhibited a weak correlation with any of the printing outcomes. This lack of correlation was unexpected and merits further investigation to understand the underlying factors influencing printing performance. One possible explanation for the weak correlation between yield stress and printing outcomes in our study is the selective inclusion of bioinks that were capable of extrusion, with the extrusion flowrate standardized across all bioinks. In a broader range of materials, such as bioinks with concentrations unsuitable for extrusion, the yield stress may exhibit stronger correlations with printing outcomes, resulting in higher R^2^ values.

Additionally, we found a poor correlation between the uniformity ratio and the rheological measures. Previous studies have suggested that tan(δ) plays a crucial role in filament uniformity, but our findings did not support this reported correlation [8,28]. It is possible that the lack of correlation could be attributed to molecular mechanisms within the polymers present in the bioink, leading to repeated entanglement and disentanglement upon shear stress, which may result in non-uniform filaments. To better observe rheological trends in printing outcomes, it may be beneficial to focus on more specific subsets of bioink classifications. For example, comparing rheological outcomes specifically for colloidal-based bioinks or suspension-based bioinks could potentially reveal more readily observable rheological trends.

The establishment of universal predictive models that apply to all bioinks remains a challenge, as the complex behavior of bioinks requires a comprehensive understanding of each rheological measure. Despite the thorough rheological characterization of bioinks, the current lack of models or equations that can accurately predict printing outcomes is evident. Future research should delve deeper into the relationships between rheological measures and the various measurements obtained from the artifact. This in-depth exploration can serve as a foundation for developing improved models with enhanced predictive capabilities. One potential direction for improvement is the incorporation of multiple rheological measures or other relevant parameters into the model. By advancing our understanding of these relationships, we can strive to develop more robust and accurate predictive models for bioink printing outcomes.

Moreover, it is essential to note that the bioinks used in practical applications are typically printed together with cell suspensions. Although this study did not include cell suspensions, we have previously investigated the impact of cell density on printing outcomes [25]. Interestingly, our findings revealed no significant differences in printing outcomes for cell densities ranging from 0 to 40 × 10^6^ cells/mL. However, slight variations in rheological properties were observed with different cell densities, indicating a potential influence on printability.

## 5. Conclusion

This study presented a comprehensive printability artifact specifically designed for bioinks, offering superior differentiation capabilities compared to other existing printability measurement approaches. The measures obtained from the artifact demonstrated reasonable accuracy and exhibited excellent discriminatory power in distinguishing between various printing outcomes. This methodology holds great potential for supporting bioink development and enhancing our understanding of the underlying factors influencing printing outcomes. While rheological measurements provided valuable insights into the shear-thinning, viscoelastic, yielding, and recovery properties of the bioink, none of these individual properties alone could fully explain the diverse range of printing outcomes observed on the artifact, although certain relationships were identified. Linear regression analysis revealed a correlation between storage modulus and the ability of unsupported bioinks to span large gaps, while tan(δ) exhibited a high correlation with turn accuracy. However, this measurement was not predictive of uniformity, except for a potential indication that low G“ values could be associated with poor uniformity. Interestingly, G” itself did not predict printing outcomes. Therefore, standardization of printability measurement techniques could be vital for bioink development. In future experiments and modeling endeavors, all these rheological properties should be considered to predict printing outcomes, mainly when aiming for general applicability across different bioinks. By incorporating a holistic understanding of these rheological properties, we can advance our ability to predict and optimize printing outcomes in bioink development accurately.

## Supplementary Material

1

2

## Figures and Tables

**Table 1:** Description of artifact measurements made from each structure. 1–18 corresponds to the respective numbers in Fig. 2.

Structure	View	Printability Aspect	Measures	Calculation
**5-Layer Tube**	Side	- Stack multiple layers	^1^Height ^2^Width	Direct measurement Direct measurement
**5-Layer Tube**	Top	- Stack multiple layers - Arc accuracy	^3^External radius ^4^Internal radius ^5^Wall thickness ^6^Radial accuracy	$r_{e}\;=\;\sqrt{A_{e}/\pi }$ $r_{i}\;=\;\sqrt{A_{i}/\pi }$ $T\;=\;r_{e}\;-\;r_{i}$ $r_{a}\;=\;\frac{(r_{e}\;+\;r_{i})/2}{4}\times 100\%$
**Crosshatch**	Top	- Form horizontal pores	^7^Pr ^8^Area of pores ^9^# of broken pores ^10^# of filled pores	$Pr\;=\;L_{p}^{2}/16A_{p}$ Direct measurement Direct measurement Direct measurement
**4-angled pattern**	Top	- Sharp turns - Single filament dimensions	^11^Filament width ^12^Standard deviation of filament width ^13^Uniformity ratio ^14^Turn angle ^15^Turn angle error	Direct measurement $\sigma_{w}\;=\;\sqrt{\frac{{\sum \left(w_{i}\;-\;\bar{w}\right)}^{2}}{N_{w}}}$ $U\;=\;\frac{(P_{1}\;+\;P_{2})/2}{L_{f}}$ Direct measurement *θ*_*e*_ = *θ*_*m*_ – *θ*_*t*_
**Overhang Collapse**	Side	- Span gaps unsupported	^16^Deflection at midpoint ^17^Angle of deflection ^18^Spanning success rate	Direct measurement *θ*_*d*_ = sin^−1^(*D*/0.5*G*) Direct measurement

Equation variables are as follows, θ_d_: angle of deflection, D: deflection at midpoint, G: gap length, r_e_: external radius, A_e_: total area contained within the external perimeter of the tube, r_i_: internal radius, A_i_: total area contained within the internal perimeter of the tube, T: wall thickness, r_a_: radial accuracy, *Pr*: Pr, L_p_: perimeter of individual pore, A_p_: area contained within the pore perimeter, σ_w_: standard deviation of filament width, w_i_: an individual width measurement,: filament width, N_w_: total number of width measurements, P_1_ and P_2_: perimeter of each side of the filament segment, L_f_: length of the filament segment, θ_e_: turn angle error, θ_m_: measured turn angle, θ_t_: theoretical/designed turn angle.

**Table 2:** Printing outcomes measured by bioink artifact quantification.

Abbr.	Bioink artifact									
	Tube height	Tube width	Wall thickness	Radial accuracy	*Pr*	Pore area	Filament width	Uniformity	Angle error	Filament deflection
**PF (standard)**	+++	+++	+++	+++	+++	+++	+++	+++	+++	+++
**GG/GM**	++	+++	+++	+++	+++	+++	+++	+	+++	+
**Alg-Lap-RD**	+++	+++	+++	+++	++	++	++	+++	+++	+++
**Alg-Lap-EP**	+++	+++	++	++	+	+	++	+++	+++	++
**ALG**	+	++	++	++	+	+	+++	+++	+	+
**MC**	+	+	+	+	**n/d**	**n/d**	**n/d**	**n/d**	**n/d**	+
**HA**	+	+	+	+	**n/d**	**n/d**	**n/d**	**n/d**	**n/d**	+

+++ Good;

++ Intermediate;

+ Poor based on the printing outcomes of PF.

n/d: not detectable.

## Data Availability

Data will be made available on request.
