# Supplementary material for: The correlation between rheological properties and extrusion-based printability in bioink artifact quantification
Source: Mater Des. Author manuscript; Available in PMC 2023 Oct 18. (PMC10583861; doi:10.1016/j.matdes.2023.112237)
Supplement: 1 [file NIHMS1936026-supplement-1.docx]

**Supplementary Information**

The correlation between rheological properties and extrusion-based printability in bioink artifact quantification

Gregory J. Gillispie^1,2^, Joshua Copus^1,2^, Meryem Uzun-Per^3^, James J. Yoo^1,2^, Anthony Atala^1,2^, Muhammad Khalid Khan Niazi^3^, and Sang Jin Lee^1,2,^*

^1^Wake Forest Institute for Regenerative Medicine, Wake Forest University School of Medicine, Winston-Salem, NC 27157, USA

^2^School of Biomedical Engineering and Sciences, Wake Forest University-Virginia Tech, Winston-Salem, NC 27157, USA

^3^Center for Biomedical Informatics, Wake Forest University School of Medicine, Winston-Salem, NC 27157, USA

**Supplementary Table 1.** Selected bioinks used in this study, their abbreviation, and the printing conditions used to print the artifact.

| **Abbr.** | **Formulation** | **Printing conditions** | | | | | |
| --- | --- | --- | --- | --- | --- | --- | --- |
|  |  | Pressure (kPa) | Flowrate (mm^3^/min) | Feedrate (mm/min) | Layer height (µm) | Nozzle size (µm) |  |
| **PF** | 40% Pluronic F127 | 258 | 84 | 150 | 420 | 330 |  |
| **GG/GM** | 1.2% gellan gum + 4% GelMA | 164 |  |  |  |  |  |
| **Alg-Lap-RD** | 1% alginate + 6% laponite RD | 140 |  |  |  |  |  |
| **Alg-Lap-EP** | 1% alginate + 6% laponite EP | 75 |  |  |  |  |  |
| **ALG** | 7% alginate | 742 |  |  |  |  |  |
| **MC** | 8% methylcellulose | 602 |  |  |  |  |  |
| **HA** | 3% hyaluronic acid | 174 |  |  |  |  |  |

**Supplementary Table 2.** All results from the 5-layer tube and crosshatch structures. a,b,c,d,e denotes statistical significance (*p*<0.05). Levels not connected by the same letter are considered significantly different.

|  |  | **Tube, side view** | | **Tube, top view** | | | | **Crosshatch pore** | |
| --- | --- | --- | --- | --- | --- | --- | --- | --- | --- |
|  |  | **Height (mm)** | **Width (mm)** | **External radius (mm)** | **Internal radius (mm)** | **Wall thickness (mm)** | **Radial accuracy (%)** | ***Pr*** | **Area (mm^2^)** |
| **PF** | Mean | 2.33 | 9.20 | 4.63 | 3.30 | 1.33 | 99.13 | 0.99 | 2.44 |
|  | St. Dev. | 0.04 | 0.03 | 0.04 | 0.01 | 0.03 | 0.58 | 0.03 | 0.02 |
|  | Sig. | b | c | b,c | a | c | a | b | a |
| **GG/GM** | Mean | 2.90 | 9.20 | 4.49 | 3.05 | 1.44 | 94.35 | 1.04 | 1.89 |
|  | St. Dev. | 0.08 | 0.19 | 0.05 | 0.04 | 0.03 | 0.99 | 0.02 | 0.32 |
|  | Sig. | a | c | c | a | c | a,b | a | b |
| **Alg-Lap-RD** | Mean | 2.35 | 9.15 | 4.52 | 3.25 | 1.27 | 97.18 | 0.92 | 1.72 |
|  | St. Dev. | 0.17 | 0.13 | 0.02 | 0.04 | 0.05 | 0.28 | 0.01 | 0.12 |
|  | Sig. | b | c | c | a | c | a | c | b |
| **Alg-Lap-EP** | Mean | 2.16 | 9.37 | 4.65 | 2.29 | 2.36 | 86.79 | 0.88 | 0.54 |
|  | St. Dev. | 0.12 | 0.10 | 0.02 | 0.06 | 0.06 | 0.93 | 0.01 | 0.14 |
|  | Sig. | b | b | b,c | b | b | c | c | c |
| **ALG** | Mean | 1.85 | 9.64 | 4.74 | 2.27 | 2.47 | 87.69 | 0.88 | 0.55 |
|  | St. Dev. | 0.03 | 0.09 | 0.02 | 0.04 | 0.02 | 0.80 | 0.01 | 0.07 |
|  | Sig. | c | b | b | b | b | b,c | c | c |
| **MC** | Mean | 1.37 | 9.11 | 4.64 | 0.00 | 4.64 | 58.01 |  |  |
|  | St. Dev. | 0.03 | 0.08 | 0.05 | 0.00 | 0.05 | 0.56 |  |  |
|  | Sig. | d | c | b,c | c | a | e |  |  |
| **HA** | Mean | 1.26 | 10.70 | 5.28 | 0.29 | 4.98 | 69.53 |  |  |
|  | St. Dev. | 0.06 | 0.18 | 0.15 | 0.42 | 0.33 | 6.63 |  |  |
|  | Sig. | d | a | a | c | a | d |  |  |

**Supplementary Table 3.** All results from the 4-angled pattern. a,b,c denotes statistical significance (*p*<0.05). Levels not connected by the same letter are considered significantly different.

|  |  | **Single filament** | | | **Turn angle (deg.)** | | | | **Turn angle error (deg.)** | | | |
| --- | --- | --- | --- | --- | --- | --- | --- | --- | --- | --- | --- | --- |
|  |  | **Width (mm)** | **Width**  **St. Dev. (mm)** | **Uniformity ratio** | **125^o^** | **90^o^** | **55^o^** | **20^o^** | **125^o^** | **90^o^** | **55^o^** | **20^o^** |
| **PF** | Mean | 1.13 | 0.020 | 1.015 | 125.6 | 88.0 | 56.6 | 21.1 | 0.55 | -2.00 | 1.59 | 1.06 |
|  | St. Dev. | 0.03 | 0.002 | 0.010 | 0.1 | 0.9 | 1.8 | 2.2 | 0.08 | 0.89 | 1.82 | 2.22 |
|  | Sig. | c | b | b | a,b | a | b,c | b | a,b | a | b,c | b |
| **GG/GM** | Mean | 1.11 | 0.114 | 1.077 | 125.5 | 90.3 | 56.8 | 19.0 | 0.50 | 0.34 | 1.79 | -1.00 |
|  | St. Dev. | 0.02 | 0.036 | 0.046 | 1.0 | 5.4 | 1.2 | 1.5 | 1.03 | 5.40 | 1.17 | 1.52 |
|  | Sig. | c | a | a | a,b | a | b,c | b | a,b | a | b,c | b |
| **Alg-Lap-RD** | Mean | 1.33 | 0.019 | 1.006 | 125.1 | 90.1 | 56.3 | 21.4 | 0.06 | 0.10 | 1.34 | 1.39 |
|  | St. Dev. | 0.11 | 0.004 | 0.003 | 0.4 | 0.9 | 0.8 | 1.0 | 0.45 | 0.95 | 0.81 | 1.00 |
|  | Sig. | a,b | b | b | b | a | c | b | b | a | c | b |
| **Alg-Lap-EP** | Mean | 1.37 | 0.050 | 1.012 | 127.7 | 86.4 | 60.1 | 20.5 | 2.74 | -3.64 | 5.08 | 0.54 |
|  | St. Dev. | 0.04 | 0.016 | 0.006 | 2.0 | 1.6 | 1.6 | 1.3 | 2.05 | 1.61 | 1.61 | 1.31 |
|  | Sig. | a | b | b | a,b | a | b | b | a,b | a | b | b |
| **ALG** | Mean | 1.20 | 0.027 | 1.011 | 128.1 | 87.4 | 65.2 | 28.3 | 3.07 | -2.58 | 10.20 | 8.32 |
|  | St. Dev. | 0.03 | 0.008 | 0.006 | 0.8 | 1.2 | 1.2 | 0.8 | 0.80 | 1.25 | 1.21 | 0.83 |
|  | Sig. | b,c | b | b | a | a | a | a | a | a | a | a |
| **MC** | Mean |  |  |  |  |  |  |  |  |  |  |  |
|  | St. Dev. |  |  |  |  |  |  |  |  |  |  |  |
|  | Sig. |  |  |  |  |  |  |  |  |  |  |  |
| **HA** | Mean |  |  |  |  |  |  |  |  |  |  |  |
|  | St. Dev. |  |  |  |  |  |  |  |  |  |  |  |
|  | Sig. |  |  |  |  |  |  |  |  |  |  |  |

**Supplementary Table 4.** All results from the overhang structure. a,b,c,d denotes statistical significance (*p*<0.05). Levels not connected by the same letter are considered significantly different.

|  |  | **Overhang deflection distance (mm)** | | | | | **Overhang deflection angle (deg.)** | | | | |
| --- | --- | --- | --- | --- | --- | --- | --- | --- | --- | --- | --- |
|  |  | **16 mm** | **8 mm** | **4 mm** | **2 mm** | **1 mm** | **16 mm** | **8 mm** | **4 mm** | **2 mm** | **1 mm** |
| **PF** | Mean | 0.71 | 0.29 | 0.17 | 0.20 | 0.13 | 5.10 | 4.16 | 4.85 | 11.66 | 15.60 |
|  | St. Dev. | 0.13 | 0.04 | 0.02 | 0.01 | 0.01 | 0.93 | 0.58 | 0.57 | 0.44 | 1.39 |
|  | Sig. | c | c | c | c | b,c | c | c | c | c | b,c |
| **GG/GM** | Mean | 2.20 | 0.83 | 0.31 | 0.26 | 0.18 | 15.96 | 11.96 | 8.89 | 14.95 | 21.13 |
|  | St. Dev. | n/a | 0.11 | 0.03 | 0.02 | 0.06 | n/a | 1.59 | 0.94 | 1.47 | 7.68 |
|  | Sig. | a | a,b | b | b,c | a,b | a | a,b | b | b,c | a,b |
| **Alg-Lap-RD** | Mean | 1.15 | 0.40 | 0.17 | 0.12 | 0.08 | 8.32 | 5.74 | 4.86 | 6.66 | 9.24 |
|  | St. Dev. | 0.14 | 0.04 | 0.01 | 0.03 | 0.00 | 0.99 | 0.51 | 0.17 | 1.78 | 0.38 |
|  | Sig. | b | c | c | d | c | b | c | c | d | c |
| **Alg-Lap-EP** | Mean | 2.25 | 0.67 | 0.36 | 0.22 | 0.18 | 16.37 | 9.68 | 10.50 | 12.49 | 20.93 |
|  | St. Dev. | 0.06 | 0.08 | 0.05 | 0.02 | 0.02 | 0.47 | 1.21 | 1.57 | 0.96 | 1.96 |
|  | Sig. | a | b | b | c | a,b | a | b | b | c | a,b |
| **ALG** | Mean |  | 0.91 | 0.48 | 0.33 | 0.23 |  | 13.09 | 13.95 | 19.35 | 27.92 |
|  | St. Dev. |  | 0.06 | 0.04 | 0.01 | 0.04 |  | 0.88 | 1.30 | 0.76 | 5.76 |
|  | Sig. |  | a | a | a | a |  | a | a | a | a |
| **MC** | Mean |  |  |  |  |  |  |  |  |  |  |
|  | St. Dev. |  |  |  |  |  |  |  |  |  |  |
|  | Sig. |  |  |  |  |  |  |  |  |  |  |
| **HA** | Mean |  |  |  | 0.30 | 0.19 |  |  |  | 17.19 | 21.90 |
|  | St. Dev. |  |  |  | 0.02 | 0.02 |  |  |  | 1.36 | 2.96 |
|  | Sig. |  |  |  | a,b | a,b |  |  |  | a,b | a,b |

**Supplementary Table 5.** All results from the rheological testing. a,b,c,d,e,f,g denotes statistical significance (*p*<0.05). Levels not connected by the same letter are considered significantly different.

|  |  | **Frequency sweep** | | **Strain sweep** | | | | **Recovery** | | | | |
| --- | --- | --- | --- | --- | --- | --- | --- | --- | --- | --- | --- | --- |
|  |  | ***n*** | ***K*** | **G' (Pa)** | **G" (Pa)** | **Tan delta** | **Yield stress (Pa)** | **Initial viscosity (Pa•s)** | **Viscosity at 3 sec (Pa•s)** | **Viscosity at 80 sec (Pa•s)** | **Recovery at 3 sec (%)** | **Recovery at 80 sec (%)** |
| **PF** | Mean | 0.2 | 11,437 | 5395.3 | 917.7 | 0.170 | 502.4 | 443.1 | 397.7 | 453.1 | 89.8 | 102.3 |
|  | St. Dev. |  |  | 176.9 | 11.6 | 0.007 | 12.3 | 11.5 | 3.4 | 16.1 | 1.6 | 4.2 |
|  | Sig. |  |  | a | a | f | b | a | a | a | b | a |
| **GG/GM** | Mean | 0.27 | 98.7 | 382.0 | 47.4 | 0.124 | 209.2 | 34.2 | 19.7 | 25.9 | 57.6 | 75.7 |
|  | St. Dev. |  |  | 38.4 | 3.6 | 0.004 | 3.9 | 0.5 | 0.9 | 0.9 | 3.4 | 3.6 |
|  | Sig. |  |  | e | g | e | d | f | f | f | e | d |
| **Alg-Lap-RD** | Mean | 0.8 | 28.3 | 4161.0 | 234.6 | 0.056 | 237.3 | 175.8 | 187.7 | 156.6 | 106.8 | 90.1 |
|  | St. Dev. |  |  | 310.5 | 11.5 | 0.002 | 4.9 | 9.2 | 11.0 | 10.0 | 2.2 | 3.6 |
|  | Sig. |  |  | b | c | g | c | c | b | c | a | b,c |
| **Alg-Lap-EP** | Mean | 0.20 | 2,755.4 | 607.9 | 152.4 | 0.251 | 18.3 | 78.2 | 39.5 | 48.1 | 50.6 | 61.6 |
|  | St. Dev. |  |  | 40.5 | 5.2 | 0.008 | 8.8 | 1.2 | 0.1 | 0.4 | 0.9 | 1.2 |
|  | Sig. |  |  | c | e | d | e | d | d | d | f | e |
| **ALG** | Mean | 0.70 | 8.46 | 434.3 | 472.8 | 1.089 |  | 227.3 | 181.3 | 197.0 | 79.8 | 86.7 |
|  | St. Dev. |  |  | 3.5 | 2.5 | 0.006 |  | 2.2 | 1.3 | 5.7 | 0.7 | 1.7 |
|  | Sig. |  |  | d | b | b |  | b | b | b | c | c |
| **MC** | Mean | 0.82 | 1,456.9 | 27.6 | 79.6 | 3.060 |  | 44.2 | 30.9 | 41.0 | 69.7 | 92.7 |
|  | St. Dev. |  |  | 11.1 | 11.3 | 0.678 |  | 0.8 | 2.3 | 1.9 | 4.5 | 3.7 |
|  | Sig. |  |  | g | f | a |  | e | e | e | d | b |
| **HA** | Mean | 0.78 | 558.9 | 309.7 | 184.1 | 0.594 |  | 183.4 | 121.1 | 161.2 | 66.0 | 87.9 |
|  | St. Dev. |  |  | 5.0 | 3.2 | 0.001 |  | 1.0 | 4.6 | 2.4 | 2.6 | 1.5 |
|  | Sig. |  |  | f | d | c |  | c | c | c | d | c |

**Supplementary Figure 1.** Linear regression analysis for (A) *n* constant, (B) *K* constant, (C) storage modulus, (D) loss modulus, (E) tan delta, (F) yield stress, and (G) recovery at 3 seconds versus wall thickness of the 5-layer tube.

**Supplementary Figure 2.** Linear regression analysis for (A) *n* constant, (B) *K* constant, (C) storage modulus, (D) loss modulus, (E) tan delta, (F) yield stress, and (G) recovery at 3 seconds versus uniformity ratio of the 4-angle pattern.
